# A Comprehensive Review on the Role of MRI in the Assessment of Supratentorial Neoplasms: Comparative Insights Into Adult and Pediatric Cases

**DOI:** 10.7759/cureus.67553

**Published:** 2024-08-23

**Authors:** Paritosh N Bhangale, Shivali V Kashikar, Paschyanti R Kasat, Priyal Shrivastava, Anjali Kumari

**Affiliations:** 1 Radiodiagnosis, Jawaharlal Nehru Medical College, Datta Meghe Institute of Higher Education and Research, Wardha, IND

**Keywords:** mri in neuro-oncology, tumor diagnosis and treatment planning, advanced imaging techniques, adult and pediatric brain tumors, magnetic resonance imaging (mri), supratentorial neoplasms

## Abstract

Magnetic resonance imaging (MRI) is a critical diagnostic tool in assessing supratentorial neoplasms, offering unparalleled detail and specificity in brain imaging. Supratentorial neoplasms in the cerebral hemispheres, basal ganglia, thalamus, and other structures above the tentorium cerebelli present significant diagnostic and therapeutic challenges. These challenges vary notably between adult and pediatric populations due to differences in tumor types, biological behavior, and patient management strategies. This comprehensive review explores the role of MRI in diagnosing, planning treatment, monitoring response, and detecting recurrence in supratentorial neoplasms, providing comparative insights into adult and pediatric cases. The review begins with an overview of the epidemiology and pathophysiology of these tumors in different age groups, followed by a detailed examination of standard and advanced MRI techniques, including diffusion-weighted imaging (DWI), perfusion-weighted imaging (PWI), and magnetic resonance spectroscopy (MRS). We discuss the specific imaging characteristics of various neoplasms and the importance of tailored approaches to optimize diagnostic accuracy and therapeutic efficacy. The review also addresses the technical and interpretative challenges unique to pediatric imaging and the implications for long-term patient outcomes. By highlighting the comparative utility of MRI in adult and pediatric cases, this review aims to enhance the understanding of its pivotal role in managing supratentorial neoplasms. It underscores the necessity of age-specific diagnostic and therapeutic strategies. Emerging MRI technologies and future research directions are also discussed, emphasizing the potential for advancements in personalized imaging approaches and improved patient care across all age groups.

## Introduction and background

Supratentorial neoplasms are tumors located in the supratentorial region of the brain, encompassing the cerebral hemispheres, basal ganglia, thalamus, and other structures above the tentorium cerebelli [1]. These neoplasms can be primary, originating within the brain tissue, or secondary, resulting from metastasis from other body parts. Primary supratentorial neoplasms include gliomas, meningiomas, pituitary adenomas, and embryonal tumors. In adults, glioblastomas and meningiomas are common, while in children, tumors such as medulloblastomas and pilocytic astrocytomas are more frequently observed [2]. The significance of these neoplasms lies in their potential impact on critical brain functions due to their location, affecting cognition, motor skills, sensory processing, and overall neurological health. Managing these tumors presents substantial challenges due to the brain structures' complexity and the necessity for precise diagnostic and therapeutic approaches [3].

Magnetic resonance imaging (MRI) is a non-invasive imaging modality that uses strong magnetic fields, radio waves, and field gradients to generate detailed images of the brain and other body parts. MRI is particularly valuable in assessing supratentorial neoplasms due to its superior soft tissue contrast, multiplanar imaging capabilities, and absence of ionizing radiation [4]. Standard MRI sequences, such as T1-weighted, T2-weighted, and fluid-attenuated inversion recovery (FLAIR), provide essential information about brain tumors' anatomical location, size, and structural characteristics. Advanced MRI techniques, including diffusion-weighted imaging (DWI), perfusion-weighted imaging (PWI), and magnetic resonance spectroscopy (MRS), offer additional insights into tumor cellularity, vascularity, and metabolic profile, respectively. These capabilities make MRI an indispensable tool for the initial diagnosis, treatment planning, and follow-up of patients with supratentorial neoplasms [5].

Differentiating between adult and pediatric cases of supratentorial neoplasms is crucial due to the significant differences in tumor types, biology, treatment responses, and prognostic outcomes. Pediatric brain tumors often differ from those in adults, not only in their histological characteristics but also in their growth patterns, genetic profiles, and responses to therapy. For instance, medulloblastomas, which are common in children, have a different biological behavior compared to glioblastomas, which are more prevalent in adults [6]. Additionally, a child's developing brain presents unique challenges in imaging and treatment. Pediatric patients may require sedation or anesthesia during MRI procedures to minimize motion artifacts, and considerations regarding the long-term effects of radiation exposure and chemotherapy on the developing brain are essential. Therefore, a tailored approach that considers these age-related differences is essential for optimizing diagnostic accuracy and therapeutic efficacy [7].

This comprehensive review aims to provide a detailed analysis of the role of MRI in assessing supratentorial neoplasms, with a comparative perspective on adult and pediatric cases. This review aims to highlight the epidemiology and pathophysiology of supratentorial neoplasms in both adult and pediatric populations and examine the various MRI techniques used in the initial diagnosis, treatment planning, monitoring treatment response, and detecting recurrence of these tumors. It will compare and contrast the MRI findings and imaging challenges between adult and pediatric cases, discuss advanced MRI techniques and their specific applications in assessing supratentorial neoplasms, and identify the challenges and limitations of MRI in this context. Additionally, the review will explore future directions in imaging technology and research to enhance the understanding of MRI's pivotal role in managing supratentorial neoplasms and underscore the importance of age-specific approaches in neuro-oncology imaging.

## Review

Basics of MRI

Magnetic resonance imaging (MRI) is a non-invasive imaging technique that uses strong magnetic fields and radio waves to produce detailed images of the body's internal structures. It is particularly beneficial for brain imaging due to its high soft tissue contrast and the absence of ionizing radiation [8]. The fundamental principles of MRI involve the behavior of hydrogen nuclei (protons) in a magnetic field. When exposed to a strong magnetic field, these protons align with it. An applied radiofrequency (RF) pulse temporarily displaces the protons from their alignment. As they realign, they emit energy in the form of detected signals and are converted into images [9]. Key components of MRI technology include the magnetic field, radiofrequency pulses, and gradient coils. The strength of the magnetic field, measured in Tesla, significantly influences image quality; clinical MRI systems typically operate at strengths between 1.5 T and 3 T. Radiofrequency pulses are essential for exciting the protons and generating the signals needed for imaging [10]. Gradient coils spatially encode the signals, enabling the creation of detailed images. Additionally, relaxation times (T1 (longitudinal relaxation) and T2 (transverse relaxation)) are crucial in determining MRI image contrast, as these times differ among various tissues [11].

**Figure 1:**
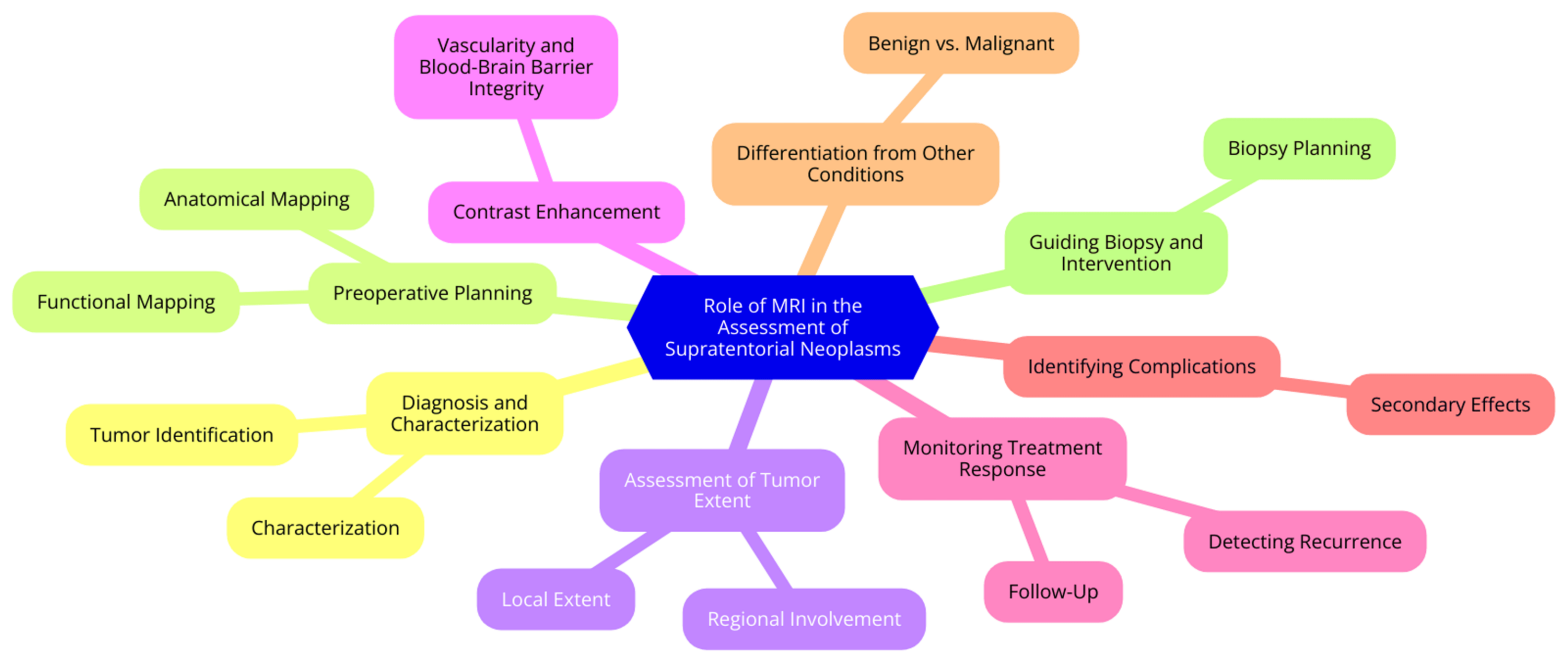
Role of MRI in the assessment of supratentorial neoplasms MRI: magnetic resonance imaging Image credit: Dr. Paritosh N. Bhangale

MRI employs a variety of sequences to optimize imaging for different clinical scenarios. Common sequences include T1-weighted imaging, useful for anatomical detail and identifying fat-containing lesions, providing high-resolution images of the brain's structure. T2-weighted imaging is particularly sensitive to edema and pathology, highlighting fluid and aiding in lesion detection [12]. Fluid-attenuated inversion recovery (FLAIR) is another important sequence that suppresses cerebrospinal fluid (CSF) signals, making visualizing lesions near the ventricles easier. Diffusion-weighted imaging (DWI) measures the movement of water molecules in tissue, providing insights into cellular integrity and is especially valuable for detecting acute ischemic strokes [13]. Advanced MRI techniques further enhance traditional MRI's diagnostic capabilities. Perfusion imaging assesses blood flow in the brain, helping evaluate tumor vascularity and overall brain perfusion in various conditions. Magnetic resonance spectroscopy (MRS) analyzes the chemical composition of tissues, offering information about metabolic changes in tumors and other brain disorders [14]. MRS can detect metabolites such as choline, creatine, and N-acetylaspartate, which indicate cellular activity and pathology [15]. MRI's role in assessing supratentorial neoplasms is shown in Figure 1.

Epidemiology and pathophysiology of supratentorial neoplasms

Supratentorial neoplasms represent a significant category of central nervous system (CNS) tumors, impacting both adult and pediatric populations. In adults, the incidence of brain tumors is estimated to be approximately 10-20 cases per 100,000 individuals annually, with supratentorial tumors accounting for about 80%-90% of all brain tumors [16]. Glioblastoma multiforme (GBM) is the most common malignant tumor in adults, followed by meningiomas and oligodendrogliomas. The male-to-female ratio for these tumors typically hovers around 1.5:1. In contrast, pediatric CNS tumors constitute about 20% of all childhood cancers, with supratentorial tumors being more prevalent than infratentorial ones [17]. The incidence in children is approximately 5-6 per 100,000 annually. Common types of supratentorial neoplasms in this demographic include pilocytic astrocytomas, medulloblastomas, and germ cell tumors, with a male-to-female ratio of about 2:1 for ependymomas and other gliomas [18].

Among adults, several types of supratentorial neoplasms are particularly noteworthy. Glioblastoma multiforme (GBM) stands out as the most aggressive and common primary brain tumor, accounting for roughly 15% of all brain tumors in this population [19]. GBM is characterized by rapid growth and a poor prognosis, with a median survival of about 15 months post-diagnosis. In contrast, meningiomas are the most common benign brain tumors and are often asymptomatic until they grow large enough to cause symptoms. These tumors typically arise from the meninges and generally have a favorable prognosis following surgical resection [17]. Oligodendrogliomas, while less common than GBM and meningiomas, often occur in the frontal and temporal lobes and are associated with better outcomes when they exhibit 1p/19q co-deletion. Additionally, astrocytomas vary in grade from low-grade (grade II) to high-grade (grade IV/GBM), with treatment and prognosis differing significantly based on the tumor grade [20].

In the pediatric population, several supratentorial neoplasms are prevalent. Pilocytic astrocytoma is a low-grade tumor commonly found in the cerebellum but can also occur supratentorially. It generally has a good prognosis and is often treated with surgical resection. Medulloblastoma, while primarily an infratentorial tumor, can present supratentorially and is a high-grade tumor more common in children, necessitating aggressive treatment [21]. Germ cell tumors, including germinomas, can occur in the supratentorial region and are particularly prevalent in males. These tumors are often sensitive to chemotherapy and radiotherapy. Ependymomas, although more common in the infratentorial region, can also occur supratentorially, especially in younger children, and exhibit distinct molecular features that influence prognosis and treatment strategies [21]. The pathophysiology of supratentorial neoplasms varies by tumor type but generally involves genetic mutations and alterations in cellular signaling pathways. Many gliomas, including GBM, are associated with mutations in key genes such as TP53 and IDH1/2, as well as chromosomal alterations (e.g., 1p/19q co-deletion in oligodendrogliomas) that drive tumorigenesis [22]. The 2016 World Health Organization (WHO) classification of CNS tumors introduced a molecular approach to categorizing gliomas, reflecting their distinct biological behavior and clinical outcomes. This classification emphasizes the importance of genetic profiling in guiding treatment strategies, highlighting the need for personalized approaches to therapy based on the specific molecular characteristics of the tumors [23].

MRI in initial diagnosis

MRI plays a crucial role in the initial diagnosis of brain neoplasms, offering essential information for detecting and characterizing tumors. Its superior soft tissue contrast allows for detailed imaging of brain structures, making it the preferred modality for identifying tumors, assessing their size, and determining the extent of infiltration into surrounding tissues [24]. Unlike other imaging techniques, MRI does not use ionizing radiation, making it a safer option for repeated imaging, particularly in pediatric populations. MRI's ability to differentiate between various tumor types and assess their characteristics, such as enhancement patterns following contrast administration, is vital for informing treatment decisions [25]. Notable differences emerge when comparing the imaging characteristics of neoplasms in adults and children. In adults, common supratentorial tumors include gliomas, meningiomas, and metastatic lesions. Gliomas typically appear hyperintense on T2-weighted images and may show heterogeneous enhancement on post-contrast images, indicating areas of necrosis or edema [26]. Meningiomas often present as well-defined extra-axial masses that enhance uniformly with contrast, displacing rather than invading adjacent brain tissue. Metastatic tumors may present as multiple lesions with variable enhancement patterns, frequently accompanied by surrounding edema [27]. In pediatric patients, brain tumors often differ in type and imaging characteristics. For instance, medulloblastomas are commonly located in the posterior fossa but can extend supratentorially. They typically appear hyperintense on T2-weighted images and show intense enhancement after contrast administration. Ependymomas usually arise from the ventricular system and can exhibit heterogeneous signal characteristics due to cystic changes or calcifications [28]. Neuroblastomas may present with supratentorial masses that invade adjacent structures, displaying varied enhancement patterns depending on their histological grade. Overall, pediatric tumors tend to be more aggressive and may present with distinct imaging features compared to adult tumors, necessitating careful interpretation of MRI findings [29].

To illustrate MRI's role in the initial diagnosis of neoplasms, consider two case examples. In an adult case, a 55-year-old male presented with headaches and focal neurological deficits. Initial MRI revealed a large, irregular mass in the right frontal lobe, which was hyperintense on T2-weighted images and showed significant surrounding edema. Post-contrast imaging demonstrated heterogeneous enhancement, suggestive of high-grade glioma, later confirmed by biopsy [30]. In a pediatric case, an eight-year-old female presented with seizures and developmental delays. MRI showed a well-circumscribed mass in the left temporal lobe, hyperintense on T2-weighted images, with a homogeneous enhancement pattern post-contrast. These imaging characteristics were consistent with a low-grade glioma, confirmed by histopathological examination following surgical resection [31].

MRI in treatment planning

Magnetic resonance imaging (MRI) has become an integral component in the treatment planning of supratentorial neoplasms, impacting various aspects such as preoperative assessment, surgical planning, biopsy techniques, radiotherapy planning, and accommodating the differences between adult and pediatric patients [32]. In preoperative assessment and surgical planning, MRI provides detailed anatomical and functional information crucial for neurosurgeons. It enables the visualization of the tumor's relationship with surrounding structures, the assessment of tumor margins, and the identification of critical areas that need preservation during surgery. High-resolution images facilitate planning surgical approaches, minimizing damage to healthy brain tissue. Advanced MRI techniques, such as diffusion tensor imaging (DTI), enhance the understanding of white matter tracts, guiding surgeons to avoid functional brain areas, thus improving surgical outcomes [33]. MRI-guided biopsy techniques leverage MRI's imaging capabilities to improve tumor sampling accuracy. These techniques allow for real-time visualization of the tumor during the biopsy procedure, ensuring that samples are taken from the most representative areas of the tumor. This is particularly beneficial when tumors are near critical structures or inaccessible. By enhancing needle placement precision, MRI guidance reduces the risk of complications and improves diagnostic yield, ultimately leading to better-informed treatment decisions [34]. MRI also plays a pivotal role in radiotherapy planning by providing superior soft tissue contrast compared to computed tomography (CT). This capability allows for better delineation of tumor volumes and surrounding healthy tissues, which is essential for effective treatment planning. Recent advancements have enabled MRI-only treatment planning, where MRI data generate pseudo-CT images for dose calculations. This approach can streamline the treatment process, reduce patient exposure to ionizing radiation, and enhance the accuracy of dose delivery. Furthermore, MRI can be integrated with real-time imaging techniques during radiotherapy, allowing for adaptive treatment strategies that adjust to changes in tumor size or patient positioning, optimizing treatment efficacy [35].

While MRI benefits adult and pediatric patients, treatment planning must consider the unique biological and anatomical differences between these populations. Adults typically present with gliomas and meningiomas, whereas pediatric patients often have embryonal tumors such as medulloblastomas. This difference influences imaging characteristics and treatment approaches. Additionally, pediatric tumors may exhibit more aggressive behavior, necessitating different treatment intensities and strategies. For instance, the tolerance of surrounding tissues to radiation may vary between adults and children, requiring careful planning to minimize long-term side effects in younger patients [36]. Moreover, MRI protocols may differ between adults and children, with pediatric protocols often tailored to reduce sedation needs and accommodate children's smaller anatomy. This necessitates adjustments in imaging parameters to ensure optimal image quality without compromising patient safety. MRI significantly enhances treatment planning for supratentorial neoplasms through detailed preoperative assessments, precise biopsy techniques, and effective radiotherapy planning. Understanding the differences in treatment approaches between adults and pediatric patients is essential for optimizing outcomes in both groups [37].

Table 1 summarizes the key points regarding MRI in treatment planning for supratentorial neoplasms.

**Table 1:** Summary of the key points regarding MRI in treatment planning for supratentorial neoplasms MRI: magnetic resonance imaging, DTI: diffusion tensor imaging, CT: computed tomography, MRS: magnetic resonance spectroscopy

Aspect	Details
Preoperative assessment	MRI provides detailed anatomical and functional information crucial for neurosurgeons, allowing visualization of tumor relationships with surrounding structures, assessment of tumor margins, and identification of critical areas.
Surgical planning	High-resolution images help in planning surgical approaches, minimizing damage to healthy brain tissue. Advanced techniques like DTI assist in understanding white matter tracts and guiding surgeons.
MRI-guided biopsy	MRI enhances the accuracy of tumor sampling by enabling real-time visualization during the biopsy procedure, ensuring samples are taken from representative areas of the tumor. It reduces the risk of complications and improves diagnostic yield.
Radiotherapy planning	MRI's superior soft tissue contrast aids in better delineation of tumor volumes and surrounding healthy tissues. MRI-only treatment planning is emerging, using MRI data to generate pseudo-CT images for dose calculations.
Integration of advanced techniques	Techniques such as DTI, perfusion MRI, and MRS are integrated into planning to enhance understanding of tumor characteristics and guide precise treatment strategies.
Considerations for pediatric patients	Pediatric protocols are often tailored to reduce sedation needs and accommodate the smaller anatomy of children, with imaging adjustments made to ensure optimal quality without compromising safety.
Age-specific challenges	Pediatric patients present unique challenges, such as more aggressive tumors and differences in radiation tolerance, requiring careful planning to minimize long-term side effects. Adults typically present with different tumor types and characteristics.

MRI in monitoring treatment response

Monitoring treatment response in patients with supratentorial neoplasms using MRI involves established criteria and advanced imaging techniques. This assessment is crucial for guiding treatment decisions, evaluating patient outcomes, and ensuring that therapies are effective, allowing for necessary adjustments [38]. The Response Evaluation Criteria in Solid Tumors (RECIST) is a widely accepted standard for measuring treatment response based on changes in tumor size. RECIST categorizes responses into four groups determined by the longest diameter of the lesions: complete response (CR), partial response (PR), stable disease (SD), and progressive disease (PD). However, the Response Assessment in Neuro-Oncology (RANO) criteria offer a more tailored approach for brain tumors. RANO considers the size of enhancing lesions, non-enhancing components, and clinical symptoms, making it particularly suitable for assessing gliomas and other central nervous system tumors. This distinction is important, as brain tumors often exhibit unique response patterns that may not be fully captured by RECIST alone [39].

Functional MRI (fMRI) techniques, such as perfusion MRI and diffusion-weighted imaging (DWI), significantly enhance the evaluation of treatment response. Perfusion MRI assesses blood flow within tumors, which can indicate treatment efficacy. Increased blood volume often correlates with tumor progression, while decreased perfusion may suggest effective treatment or necrosis. On the other hand, DWI provides insights into the cellularity of tumors. Changes in the apparent diffusion coefficient (ADC) values can help distinguish between true progression and treatment-related changes, such as necrosis or pseudoprogression. This distinction is particularly relevant in immunotherapy, where treatment effects can sometimes mimic tumor growth [40].

The response to treatment and the interpretation of MRI findings can differ significantly between adult and pediatric patients. Adults typically present with a higher incidence of high-grade tumors, which may exhibit more aggressive behavior and complex treatment responses. In this demographic, using advanced imaging techniques such as perfusion MRI is critical to accurately assess treatment effects and guide subsequent therapy. Conversely, pediatric patients often have distinct tumor types, such as medulloblastomas and low-grade gliomas, which may respond differently to treatment. The biological behavior of these tumors can lead to different imaging characteristics, necessitating a tailored approach to interpreting MRI results [41].

Table 2 summarizes the key points regarding MRI in monitoring treatment response for supratentorial neoplasms.

**Table 2:** Summary of the key points regarding MRI in monitoring treatment response for supratentorial neoplasms MRI: magnetic resonance imaging, RECIST: Response Evaluation Criteria in Solid Tumors, RANO: Response Assessment in Neuro-Oncology, DWI: diffusion-weighted imaging, ADC: apparent diffusion coefficient

Aspect	Details
Response evaluation criteria	Utilizes criteria such as RECIST for measuring treatment response based on changes in tumor size. The RANO criteria are more tailored for brain tumors, considering enhancing lesions, non-enhancing components, and clinical symptoms.
Functional MRI techniques	Functional MRI techniques such as perfusion MRI and DWI enhance treatment response evaluation by providing insights into tumor blood flow and cellularity. Perfusion MRI assesses blood volume, while DWI measures the ADC values to distinguish between true tumor progression and treatment-related changes.
Differences in adult and pediatric patients	Treatment response and MRI interpretation can differ between adults and children. Adults often present with high-grade tumors requiring more aggressive imaging techniques. Pediatric patients, who commonly have tumors such as medulloblastomas, may respond differently, necessitating a tailored approach.
Challenges in interpretation	Distinguishing between true tumor progression, pseudoprogression, and treatment-related changes (e.g., necrosis) is crucial. Advanced MRI techniques help in accurate differentiation, but sometimes, histopathological confirmation through biopsy may be required.
Importance of follow-up scans	Regular follow-up MRI scans are essential to monitor treatment response and detect recurrence. These are typically scheduled at regular intervals (e.g., every 3-6 months for the first two years post-treatment), with standardized imaging protocols ensuring consistency.

MRI in detecting recurrence

Magnetic resonance imaging (MRI) is a critical tool in detecting recurrence in supratentorial neoplasms. Its ability to provide detailed images of brain structures makes it invaluable for differentiating between tumor recurrence and treatment-related changes. This review discusses the techniques employed, the importance of follow-up MRI protocols, and comparative insights into recurrence detection in adults and children [42]. Differentiating tumor recurrence from treatment-related changes, such as radiation necrosis or pseudoprogression, is challenging. Advanced MRI techniques have been developed to enhance diagnostic accuracy. Diffusion-weighted imaging (DWI) assesses the movement of water molecules in tissue. Recurrent tumors typically exhibit high cellularity, resulting in lower apparent diffusion coefficient (ADC) values than treatment effects, which show higher ADC values due to necrosis and edema. Additionally, MRI perfusion techniques measure relative cerebral blood volume (rCBV) [43]. Recurrent high-grade gliomas (HGG) often present with elevated rCBV, while radiation effects tend to show lower rCBV values. Magnetic resonance spectroscopy (MRS) can also distinguish between recurrence and treatment effects by analyzing metabolic changes, for example, elevated choline (Cho) levels relative to creatine (Cr) in recurrent tumors contrast with lower Cho levels in areas of necrosis or treatment effect. These advanced imaging modalities significantly improve the ability to differentiate between true tumor recurrence and benign post-treatment changes, although definitive diagnosis may still require histopathological confirmation through biopsy [44].

Regular follow-up MRI is essential for monitoring patients post-treatment. Establishing a consistent MRI protocol helps in the early detection of recurrence, which is crucial for timely intervention. Key considerations include the timing of follow-up scans, typically scheduled at regular intervals, such as every 3-6 months for the first two years post-treatment and then annually, depending on the individual risk of recurrence [45]. Standardized imaging protocols are also important; consistency in imaging sequences and parameters across follow-up scans allows for better comparison over time, helping to identify subtle changes that may indicate recurrence. Furthermore, MRI findings should always be interpreted with clinical symptoms and other diagnostic modalities to ensure comprehensive evaluation and appropriate management of recurrence [46]. The recurrence patterns and MRI's effectiveness in detecting these recurrences can differ significantly between adults and children. In adults, recurrent gliomas are more prevalent, and MRI is often the first-line imaging modality for surveillance. Studies indicate that adult recurrence can manifest as new enhancing lesions or changes in existing lesions, which may be subtle and require careful interpretation of advanced imaging techniques [47]. In contrast, in children, the types of supratentorial neoplasms often differ, with a higher incidence of low-grade gliomas and primitive neuroectodermal tumors (PNETs). MRI remains crucial for detecting recurrence in pediatric cases, but the imaging characteristics may vary. For instance, pediatric tumors may present with more pronounced edema and different enhancement patterns compared to adult tumors, necessitating tailored imaging approaches [48].

Advanced MRI techniques

Advanced MRI techniques, including magnetic resonance spectroscopy (MRS), functional MRI (fMRI), and diffusion tensor imaging (DTI), play crucial roles in the assessment of neoplasms. These modalities offer insights into metabolic activity, functional impacts on the brain, and white matter integrity, providing a comprehensive understanding of tumor characteristics and their effects on surrounding brain structures [49]. Magnetic resonance spectroscopy (MRS) is a powerful, non-invasive tool that provides biochemical profiles of brain tumors. It allows for assessing metabolites such as choline, creatine, and N-acetylaspartate (NAA), critical in understanding tumor biology and behavior. Proton MRS (1H-MRS), the most commonly used form, can help differentiate tumor types, assess histological grades, and monitor treatment responses [50]. Emerging techniques such as three-dimensional echo planar spectroscopic imaging (3D-EPSI) and chemical exchange saturation transfer (CEST) are enhancing the spatial resolution and metabolic mapping capabilities of MRS, enabling better delineation of tumor margins and infiltration patterns. This metabolic assessment is particularly valuable in distinguishing between tumor recurrence and treatment-related changes, thereby guiding clinical decision-making [51]. Functional MRI (fMRI) assesses brain activity by measuring changes in blood flow, providing insights into how tumors affect surrounding brain function. This is particularly important in surgical planning, as it helps identify critical functional areas that must be preserved during tumor resection. In both adults and children, fMRI can reveal alterations in language, motor, and cognitive functions due to tumor presence. This technique is vital for tailoring treatment plans that minimize functional deficits post-surgery. For instance, in cases where tumors are located near eloquent areas of the brain, fMRI can guide neurosurgeons in planning their approach to avoid damaging essential neural pathways [52].

Diffusion tensor imaging (DTI) is an advanced MRI technique that evaluates the integrity of white matter tracts in the brain. By measuring the diffusion of water molecules in different directions, DTI provides information about the microstructural integrity of white matter, which can be compromised by tumor infiltration. DTI is particularly beneficial in assessing the extent of infiltration in high-grade gliomas and other aggressive tumors. It can help visualize changes in white matter pathways, guide surgical approaches, and predict functional outcomes in adult and pediatric patients. The ability to map these pathways is crucial for understanding how tumors may affect cognitive and motor functions, particularly in younger patients whose brains are still developing [53]. While the underlying principles of these advanced MRI techniques remain consistent across age groups, their applications can differ significantly. In adult populations, the focus is often on high-grade tumors, where MRS provides essential information for treatment planning and prognostication. For example, elevated choline levels may indicate a higher-grade tumor, prompting more aggressive management strategies. fMRI is crucial for preserving functional areas during surgery, especially in complex cases involving eloquent brain regions, where the risk of postoperative deficits is a significant concern [49]. In pediatric cases, the spectrum of brain tumors often includes different types, such as medulloblastomas and low-grade gliomas. Here, MRS can assist in differentiating these tumors based on their metabolic profiles, while fMRI helps assess developmental impacts on cognitive functions. DTI is particularly useful in evaluating the impact of tumors on developing white matter pathways, which can influence long-term outcomes and cognitive development in children. Visualizing these pathways allows clinicians to make informed decisions regarding the timing and type of interventions needed [54].

Table 3 summarizes the key points regarding advanced MRI techniques used in the assessment of supratentorial neoplasms.

**Table 3:** Summary of the key points regarding advanced MRI techniques used in the assessment of supratentorial neoplasms MRI: magnetic resonance imaging, MRS: magnetic resonance spectroscopy, NAA: N-acetylaspartate, 3D-EPSI: three-dimensional echo planar spectroscopic imaging, CEST: chemical exchange saturation transfer, fMRI: functional MRI, DTI: diffusion tensor imaging

Technique	Details
MRS	MRS provides biochemical profiles of brain tumors by assessing metabolites such as choline, creatine, and NAA. It is used to differentiate tumor types, assess tumor grades, and monitor treatment response. Emerging techniques such as 3D-EPSI and CEST enhance spatial resolution and metabolic mapping.
fMRI	fMRI measures brain activity by detecting changes in blood flow, offering insights into how tumors affect surrounding brain function. It is crucial in surgical planning to preserve critical functional areas (e.g., language and motor regions), especially when tumors are near eloquent brain regions.
DTI	DTI evaluates the integrity of white matter tracts by measuring the diffusion of water molecules in different directions. It is particularly useful for visualizing white matter pathways affected by tumor infiltration, guiding surgical approaches, and predicting functional outcomes.
Perfusion MRI	Perfusion MRI assesses blood flow within tumors, providing information on tumor vascularity. It helps differentiate between tumor types and stages and can be used to evaluate treatment effectiveness by monitoring changes in tumor blood flow.
Challenges and applications	Advanced MRI techniques require specialized knowledge for accurate interpretation. While underlying principles are consistent across age groups, their application can vary significantly between adult and pediatric patients, particularly in understanding tumor behavior and planning interventions.
Role in personalized medicine	These techniques contribute to personalized imaging approaches, enabling tailored diagnostic and therapeutic strategies based on the unique characteristics of each tumor and patient. They are increasingly important in refining treatment plans and improving patient outcomes.

Challenges and limitations

While MRI is a powerful tool for assessing supratentorial neoplasms, several challenges and limitations impact its effectiveness, especially when comparing adult and pediatric cases. Understanding these challenges is crucial for optimizing imaging strategies and improving diagnostic accuracy [5]. A major technical challenge in pediatric MRI is the need for sedation in younger children. Many pediatric patients struggle to remain still during the scan, leading to motion artifacts and suboptimal imaging quality. Sedation itself poses additional risks, including respiratory complications and prolonged recovery times. Consequently, careful consideration is required to balance the need for sedation with its potential impact on imaging quality [37]. Additionally, the developmental stage of pediatric patients can limit their cooperation during the procedure. Children may experience anxiety or fear, which complicates the imaging process. While child-friendly environments and techniques are employed to reduce stress, they may still result in incomplete or inadequate imaging [55]. Interpreting MRI findings in pediatric patients is further complicated by significant developmental differences in the brain. The pediatric brain undergoes substantial changes from infancy through adolescence, affecting the appearance of normal anatomical structures on MRI. Radiologists must recognize normal variations in pediatric brain anatomy to avoid misdiagnosis [56]. Moreover, tumors in children may present differently from those in adults, with some exhibiting atypical imaging characteristics. For instance, low-grade gliomas in children may appear less aggressive on MRI, potentially leading to an underestimation of their clinical significance. A thorough understanding of typical and atypical presentations in pediatric populations is essential [57].

Another limitation of MRI in assessing supratentorial neoplasms is the challenge of differentiating tumor types based solely on imaging characteristics. MRI findings can overlap among different tumor types, making it difficult to distinguish between them. For example, gliomas and metastases may show similar enhancement patterns and edema [58]. This limitation underscores the importance of integrating clinical information, histopathological data, and advanced imaging techniques, such as MRS, to achieve accurate diagnoses. A definitive diagnosis may often require a biopsy or surgical intervention, particularly when imaging characteristics are ambiguous. This is especially pertinent in pediatric cases, where the risks of surgical intervention must be carefully weighed against the benefits of obtaining a definitive diagnosis [59]. Comparing limitations in adult and pediatric imaging reveals unique challenges for both demographics. In adults, while MRI is effective in characterizing tumors, distinguishing between primary brain tumors and metastatic lesions can be challenging, particularly in patients with a history of cancer. Age-related changes in brain structure can also confound tumor assessment in older adults. In pediatric cases, technical and interpretative challenges are compounded by the rarity of certain tumor types and their variable presentation. Furthermore, the limited availability of pediatric-specific imaging protocols can lead to inconsistencies in imaging quality and interpretation [60].

Table 4 summarizes the key points regarding the challenges and limitations of MRI in the assessment of supratentorial neoplasms.

**Table 4:** Summary of the key points regarding the challenges and limitations of MRI in the assessment of supratentorial neoplasms MRI: magnetic resonance imaging

Challenge/limitation	Details
Need for sedation in pediatric patients	Young children often require sedation to remain still during MRI scans, which poses risks such as respiratory complications and prolonged recovery times. Sedation may also affect the imaging quality due to potential motion artifacts.
Developmental differences in pediatric brain	The pediatric brain undergoes significant developmental changes, affecting the appearance of normal anatomical structures on MRI. This complicates the interpretation of MRI findings, as radiologists must differentiate between normal variations and pathological changes.
Differentiation of tumor types	MRI findings can overlap among different tumor types, making it difficult to distinguish between them based solely on imaging characteristics. For example, gliomas and metastatic lesions may appear similar, necessitating the integration of clinical information and advanced imaging techniques.
Interpreting MRI in the context of treatment effects	Differentiating between tumor recurrence and treatment-related changes (e.g., radiation necrosis and pseudoprogression) is challenging. Advanced MRI techniques help, but sometimes, histopathological confirmation is required for accurate diagnosis.
Technical limitations	MRI machines have limitations in terms of resolution and signal-to-noise ratio, which can affect the quality of the images. In some cases, artifacts or technical limitations may obscure critical details, impacting diagnostic accuracy.
Age-specific imaging protocols	The need for age-specific imaging protocols can lead to inconsistencies in imaging quality and interpretation. Pediatric-specific protocols may be limited, leading to challenges in obtaining optimal images without compromising patient safety.
Availability and accessibility	Advanced MRI techniques and pediatric-specific protocols may not be readily available in all healthcare settings, leading to disparities in diagnostic and treatment planning capabilities, especially in underserved regions.
Cost- and resource-intensive	MRI, particularly advanced techniques, can be expensive and resource-intensive, limiting their accessibility and use in routine clinical practice. This is especially challenging in resource-limited settings where cost-effective alternatives may be necessary.

Future directions

As MRI technology continues to evolve, several emerging trends and advancements hold promise for enhancing the assessment of supratentorial neoplasms in adult and pediatric populations. Advanced imaging techniques, such as diffusion-weighted (DWI) and functional MRI (fMRI), are increasingly important. These methods offer detailed insights into tissue integrity and brain function, allowing clinicians to obtain comprehensive and precise information to improve diagnosis and treatment planning [36]. Additionally, integrating artificial intelligence (AI) into MRI processes will revolutionize the field. AI algorithms can analyze imaging data more efficiently, leading to faster and more accurate diagnoses. This advancement could also reduce costs by streamlining workflows and minimizing the need for repeat scans [36]. Research trends are shifting toward a more proactive approach, focusing on how emerging technologies will influence clinical workflows and healthcare professionals' roles in MRI assessments. Investigating the impact of these advancements is crucial for anticipating changes in practice and ensuring that clinicians are prepared for future developments. Moreover, reflecting on the responsibilities of healthcare providers through the co-creation of imaginaries can help make uncertain futures more tangible, fostering greater awareness of the implications of technologies such as quantitative MRI [61]. Personalized imaging approaches are also likely to play a significant role in the future of MRI assessments for supratentorial neoplasms. Developing smaller, more affordable, accessible MRI systems could enable their use in wider healthcare settings, including smaller hospitals and clinics. This increased accessibility would enhance the affordability of MRI scans, particularly in underserved areas. Additionally, novel MRI platforms that are less prone to artifacts could open new avenues for clinical applications, such as improved imaging of implants or lung conditions. These advancements will allow for more tailored diagnostic capabilities, catering to the specific needs of individual patients [62].

## Conclusions

In conclusion, MRI is indispensable in the comprehensive assessment of supratentorial neoplasms, offering detailed insights crucial for accurate diagnosis, effective treatment planning, and ongoing monitoring. This review has highlighted the unique characteristics and challenges associated with MRI in adult and pediatric populations, emphasizing the need for tailored diagnostic approaches that consider the distinct biological and anatomical differences between these groups. Advanced MRI techniques, such as diffusion-weighted imaging, perfusion-weighted imaging, and magnetic resonance spectroscopy, have significantly enhanced our ability to evaluate tumor characteristics and treatment responses. However, challenges remain, particularly in differentiating between tumor recurrence and treatment-related changes and managing the technical and interpretative complexities of the developing pediatric brain. Future advancements in MRI technology and ongoing research are poised to refine our understanding and management of supratentorial neoplasms, ultimately improving patient outcomes through more precise and personalized care strategies. This evolving landscape underscores the critical importance of continued innovation and specialization in neuro-oncology imaging.
